# Melatonin Pretreatment Enhances the Homing of Bone Marrow-derived Mesenchymal Stem Cells Following Transplantation in a Rat Model of Liver Fibrosis

**DOI:** 10.7508/ibj.2016.04.004

**Published:** 2016

**Authors:** Keywan Mortezaee, Parichehr Pasbakhsh, Iraj Ragerdi Kashani, Fatemeh Sabbaghziarani, Ameneh Omidi, Adib Zendedel, Soudabeh Ghasemi, Ahmad Reza Dehpour

**Affiliations:** 1 Department of Anatomy, School of Medicine, Tehran University of Medical Sciences, Tehran, Iran; 2 Institute of Neuroanatomy, School of Medicine, RWTH Aachen University, 52074 Aachen, Germany; 3 Department of Pharmacology, School of Medicine, Tehran University of Medical Sciences, Tehran, Iran

**Keywords:** Bone marrow, Mesenchymal stem cells, Melatonin

## Abstract

**Background:**

Bone marrow-derived mesenchymal stem cells (BMMSCs) transplantation has been considered as a promising milestone in liver fibrosis treatment. However, low amounts of homing are a major obstacle. We aimed to investigate the role of melatonin pretreatment in BMMSC homing into experimental liver fibrosis.

**Methods:**

BMMSCs were obtained, grown, propagated and preconditioned with 5 µM melatonin and analyzed for multipotency and immunophenotypic features at passage three. The cells were labelled with CM-Dil and infused into the rats received the i.p. injection of carbon tetrachloride (CCl_4_) for five weeks to induce liver fibrosis. Animals were divided into two groups: One group received BMMSCs, whereas the other group received melatonin-pretreated BMMSCs (MT-BMMSCs). After cell injection at 72 h, animals were sacrificed, and the liver tissues were assessed for further evaluations: fibrosis using Masson’s trichrome and hematoxylin and eosin staining and homing using fluorescent microscopy and flow cytometry.

**Results:**

BMMSCs and MT-BMMSCs expressed a high level of CD44 but low levels of CD11b, CD45 and CD34 (for all *P*≤0.05) and were able to differentiate into adipocytes and Schwann cells. CCl_4_ induction resulted in extensive collagen deposition, tissue disruption and fatty accumulation with no obvious difference between the two groups. There was a significant increase in homing of MT-BMMSCs in both florescent microscopy (*P*≤0.001) and flow cytometry (*P*≤0.01) assays, as compared with non-treated BMMSCs.

**Conclusion:**

This study indicates the improved homing potential of BMMSCs in pretreatment with melatonin. Therefore, this strategy may represent an applied approach for improving the stem cell therapy of liver fibrosis.

## INTRODUCTION

In spite of great scientific breakthroughs toward treatment protocols, there are still a huge number of people suffer from incurable diseases around the world. Mesenchymal stem cells (MSCs) could be a promising source of therapy for patients to achieve their normal life[1]. Some standard criteria have been presented for MSCs, including plastic adherence, CD marker expression and multilineage differentiation. MSCs express CD44 at the high level and CD11b, CD34 and CD45 at the low levels; these cells have the capability to differentiate into adipocytes and Schwann cells[2,3]. MSCs constitute approximately 0.001% to 0.01% of all bone marrow cells[4].

Bone marrow-derived MSCs (BMMSCs) are an expandable population of stem cells with the capability of self-renewal[4] and are considered to have a potential in stem cell-based therapy[5]. Evidence has suggested the contribution of BMMSCs in regression of liver fibrosis induced by carbon tetrachloride (CCl_4_) as a well-known animal model[6]. The fact is that the use of MSCs for cell therapies depends on the capability of these cells to home into the damaged area[1]. Local injection of MSCs could be advantageous in certain aspects; however, there are special interests toward systemic infusion of MSCs with the possibility of minimal invasiveness. A predominant obstacle to the efficacious implementation of MSCs therapy is the inability to target these cells to organs of interests with high efficacy[7,8] because of oxidative stress, inflammation and hypoxia[9]. It might be presumed that increasing the number of injected cells could compensate the incidence of homing. However, this technique might not be helpful due to disturbing blood flow and demanding long-term cultivation, which in turn may change their MSCs properties and could be unsuitable for clinical usage[1]. Therefore, it is essential to pursue the strategy of *ex vivo* handling for better therapeutic outcome[9].

The pineal gland hormone melatonin (N-acetyl-5-methoxytryptamine) is known for its antioxidant activity[10] as well as MSCs survival[9] and motility[11]. Its preconditioning application in BMMSCs has been applied so far in targeting cerebral[12] and renal[9] ischemia and also myocardial infarction[13]. The purpose of the present study is to evaluate whether melatonin pretreatment could improve the mentioned significant barrier, namely the lack of homing to the efficacious implementation of MSC-based therapy in the fibrotic liver model.

## MATERIALS AND METHODS

### Cell isolation and culture

Under sterile conditions, the femurs and tibias of six-week male Sprague-Dawley rats were excised. The excision was carried out with special attention to remove all connective tissue attached to bones. The ends of the bones were cut, and bone marrow cells were obtained by carefully flashing DMEM (Gibco, USA) using a 23G syringe. The cells were then centrifuged (Hettich, Germany) at 224×g for 5 min, plated onto 25-cm^2^ culture flasks and cultured in an amplification medium, i.e. a low-glucose DMEM supplemented with 15% FBS (Gibco, USA) and 100 U/ml penicillin and 100 μg/ml streptomycin(Gibco, USA). Cells were incubated at 37°C in a humidified atmosphere of 5% CO_2_. Medium was replaced after 24 h and once every three days thereafter. The isolation of mesenchymal population was based on their capability to adhere to the culture plate[2]. At 90% confluence, the cells were recovered by digestion with 0.25% trypsin-EDTA (Gibco, USA) for 5 min and passaged to 25-cm^2^ flasks at 1:2 ratios and observed under a phase-contrast microscope (Olympus CKX41, Japan) for evaluation of morphologic features. For further experiments, passage-three cells were used.

### Melatonin pretreatment of mesenchymal stem cells

To determine the effect of melatonin on BMMSCs, cells were subjected to a 24-h pretreatment with 5 µM melatonin (Sigma, USA). Melatonin-pretreated BMMSCs (MT-BMMSCs) were then washed three times with PBS (Sigma, USA) for complete removal of the hormone from cell suspension[9,12].

### Bone marrow-derived mesenchymal stem cells multilineage differentiation potential

#### Adipogenic induction

For adipogenic differentiation, cells were incubated in an adipogenic medium containing 0.5 mM isobutylmethylxanthine, 0.5 µM dexamethasone, and 50 µM indomethacin (all from Sigma, USA) for 21 days with a medium exchange of every 3-4 days. Adipogenesis was measured by the accumulation of neutral lipids in vacuoles and stained with Oil Red O.

#### Transdifferentiation of Schwann cells

After sub-culturing at the concentration of 10^6^ cells/cm^2^, BMMSCs and MT-BMMSCs were incubated in a serum-free DMEM containing 1 mM beta-mercaptoethanol (Sigma, USA) for 24 h. Then the culture media were replaced by DMEM containing 10% FBS and 35 ng/ml all-trans-retinoic acid (Sigma, USA) for three days. Finally, for further 10 days, the cells were placed in an inducer medium containing DMEM, 10% FBS and trophic factors, including 5 ng/ml platelet-derived growth factor (Peprotech, UK), 10 ng/ml beta fibroblast growth factor (Peprotech, UK), 5 µM forskolin (Calbiochem, Canada) and 200 ng/ml hergulin (R&D Systems, USA).

#### Immunostaining of cultured cells in Schwann cell transdifferentiation medium

Differentiated cells cultured on chamber slides (Lab-Tek, Denmark) were fixed in 4% (w/v) paraformaldehyde at 4°C for 20 min. The cells were incubated with primary antibody to S100 (rabbit polyclonal; 1:200; Dako, Denmark) at 4°C overnight. On the next day, the slides were incubated with fluorescein isothiocyanate(FITC)-conjugated secondary antibody (horse anti-mouse or goat anti-rabbit; 1:100; Vector Labs., USA) at room temperature for 2 h. Cell nuclei were labeled with Hoechst dye (Sigma, USA) at room temperature for 60 min, and the slides were examined under a fluorescent microscope (Olympus BX51TRF, Japan).

### Characterization of bone marrow-derived mesenchymal stem cells

Cells, with or without melatonin preconditioning, were characterized by flow cytometric analysis of specific surface antigens. The cells were harvested with 0.25% Trypsin-EDTA, centrifuged at 112×g for 5 min, suspended in PBS and incubated in the dark with FITC-conjugated monoclonal antibodies against CD11b, CD45, CD34 and CD44 (all from eBioscience, CA, USA)— 1:100 dilution— at 4ºC for 30 min. The cells were then washed, resuspended in PBS and analyzed by flow cytometry (FACSCalibur, BDBiosciences, USA).

### CM-Dil labeling of pretreated cells

BMMSCs and MT-BMMSCs were harvested by trypsinization and suspended in 1 ml PBS containing 4 µM fluorescent dye CM-Dil (C7000, Molecular Probes™, USA). Next, the cells were incubated in a humidified CO_2_ incubator (5% CO_2_/95% air) at 37°C for 15 min, followed by a centrifugation (at 112×g for 5 min) to remove unincorporated dye. The labelled cells were rinsed, resuspended in 1 ml PBS and kept in the dark before transplantation.

### Animals

In total, 24 adult male Sprague-Dawley rats (Pasteur Institute of Iran, Tehran), weighing 200-220 g, were housed in groups of four in stainless steel cages. The animals were subjected to control conditions of constant humidity (55-65%), temperature (22°C) and illumination (12 h light-dark cycle), provided with unlimited access to food and water. The rats were allowed to acclimatize for at least one week before use. All animal experiments were carried out in accordance with the guidelines of Ethical Committee of Tehran University of Medical Sciences corresponding to the national and institutional guidelines for animal care and use.

### Carbon tetrachloride-induced fibrotic rat model and cell transplantation

Induction of liver fibrosis was performed by i.p. administration of CCl_4_ (1 ml/kg body weight, Sigma, USA) dissolved in olive oil (Sigma, USA) at a ratio of 1:1 twice a week for 5 weeks. Twenty four hours after the 10^th^ injection of CCl_4_, 1.5×10^6^ BMMSCs (CM-Dil-labelled) were injected via tail vein. For examination of the homing rate and histological stainings, rats were randomly divided into two groups of eight animals: Group I received BMMSCs, while group II received MT-BMMSCs. Animals were killed 72 h after the cell injection. Moreover, eight intact rats were used as control for histological assessments.

### Histological analysis

Liver specimens were collected, fixed in 10% neutral buffered formalin, embedded in paraffin, and cut into 5-µm sections. The slides were then heated in a vacuum oven at 60°C for 30 min and deparaffinized. The sections were stained with hematoxylin and eosin and Masson’s trichrome for structural and fibrosis detection and observed under a light microscope (Olympus CX31, Japan).

### Homing assay

#### Florescent microscopic study

After permeabilization with 0.2% Triton X-100 (Merck, Germany) and subsequent washing with PBS, deparaffinized sections of 5-μm thickness (three slides from each liver samples) were incubated with Hoechst dye under dark conditions at room temperature for 30 min to label the nuclei. The slides were visualized with an Olympus BX51 microscope (Olympus, Tokyo, Japan) equipped with an E.30 digital camera (Olympus, Tokyo, Japan). The average intensity (the average pixel value in the fluorescent stained cells) was measured in 10 high-power fields of each section with ImageQuant software (TotalLab Quant, UK) based on the area value, which is the number of pixels in the stained cells. Briefly, all the whole specific fluorescent areas of CM-Dil-positive cells were drawn in the software, the resulting table of data was exported to Excel, and all the fluorescent area values were sum up. Then, the number was divided by the area of the image and multiplied by 100.

#### Flow cytometry

The identification of homing efficiency was assayed by flow cytometry. For this purpose, rats were perfused with normal saline 72 h after transplantation, liver samples were finely smashed by forceps and placed in a tube containing collagenase IV (Invitrogen, USA). The resulting suspension was passed through 150-μm mesh, centrifuged (134×g, 5 min), exposed to 1 ml lysis buffer and eventually suspended in 1 ml PBS for further analysis of fluorescence intensity using flow cytometry. The samples were conducted in triplets and evaluated for red fluorescence CM-Dil at 570 nm. The percentage of the transplanted labeled cells homed to liver was determined as follows: %homing=[(A×B)/C] ×100%, where A is the percent of Dil cells analyzed by flow cytometry, B is the total number of cells transplanted, and C is the whole organ cellularity[14].

### Statistical analysis

The results were expressed as the mean±SD. The significant differences between the groups were evaluated using independent samples *t*-test using SPSS16 computer software. *P*≤0.05 was considered to be statistically significant.

## RESULTS

### Bone marrow-derived mesenchymal stem cells morphologic feature

**Fig. 1:**
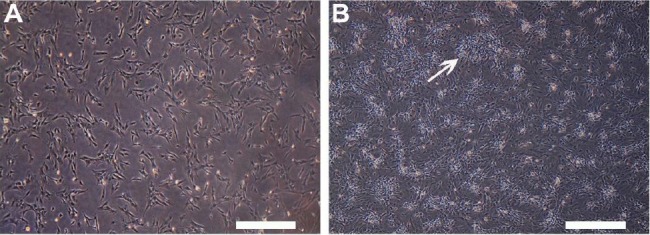
Morphologic features of bone marrow-derived mesenchymal stem cells (BMMSCs). A) Cultured BMMSCs showing spindle-shaped fibroblastic morphology; B) BMMSCs formed colonies (arrow) and became confluent (Phase-contrast microscope, scale bar = 100 µm).

A single-step primary rat BMMSCs purification method was employed using adhesion to cell culture plastic[15]. Phase-contrast microscopy revealed that the adherent cells had a spindle-shaped fibroblastic morphology (Fig. 1A), which began to form colonies and became confluent (Fig. 1B).

### Multilineage differentiation potential

#### Adipogenic differentiation

**Fig. 2:**
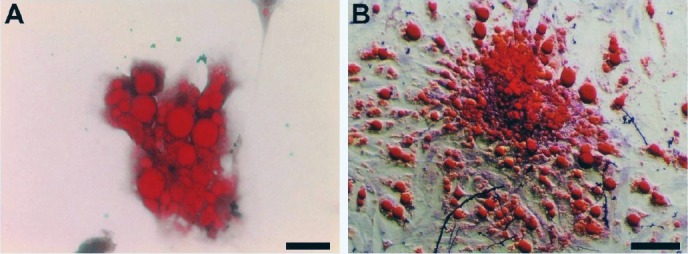
Adipogenic differentiation potential of bone marrow-derived mesenchymal stem cells (BMMSCs) stained with Oil Red O. A) By culturing in adipogenic medium, melatonin-pretreated BMMSCs exhibited a spherical shape and red-stained intracellular fat deposits; B) Non-treated BMMSCs represented the same feature as A (Light microscope, scale bar=50 µm).

BMMSCs were cultured under adipogenic condition to examine whether the cells have the differentiation capability. After three weeks of exposure to adipogenic supplementation, Oil Red O-stained BMMSCs and MT-BMMSCs showed morphological changes from fibroblastic to spherical and the accumulation of red-stained intracellular fat deposits similar to differentiated adipocytes. However, there were no apparent differences between the two groups of the cells (Fig. 2).

#### Immunocytochemistry of Schwann cell trans-differentiation of bone marrow-derived mesenchymal stem cells

**Fig. 3:**
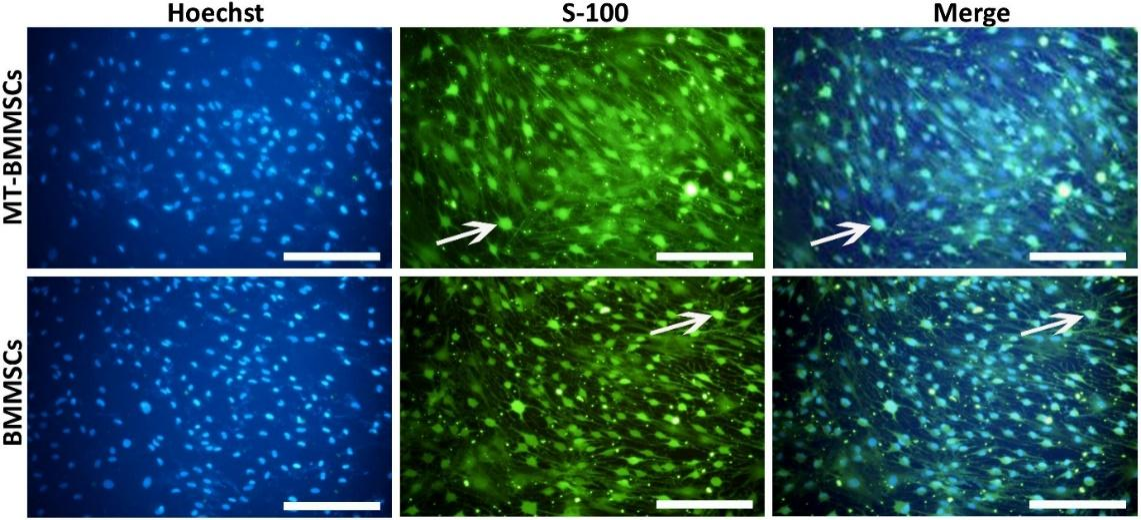
Immunocytochemistry of Schwann cell marker, S-100, in bone marrow-derived mesenchymal stem cells (BMMSCs) after 14 days of placing in transdifferentiation medium. Subsequent to the induction, differentiated melatonin-pretreated BMMSCs and BMMSCs became positive for S-100 antibody (arrows). Hoechst was used for highlighting nuclei (blue). (Fluorescent microscope, scale bar=100 µm).

To evaluate the nature of Schwann cells, we detected the expression of Schwann cell marker S-100. Almost all the transdifferentiated BMMSCs and MT-BMMSCs were positive to the S-100 antibody, and after 14 days of induction, the expression of S-100 protein was demonstrated in the cytoplasm of Schwann cells (Fig. 3).

### Immunophenotypic characterization of bone marrow -derived mesenchymal stem cells

**Fig. 4:**
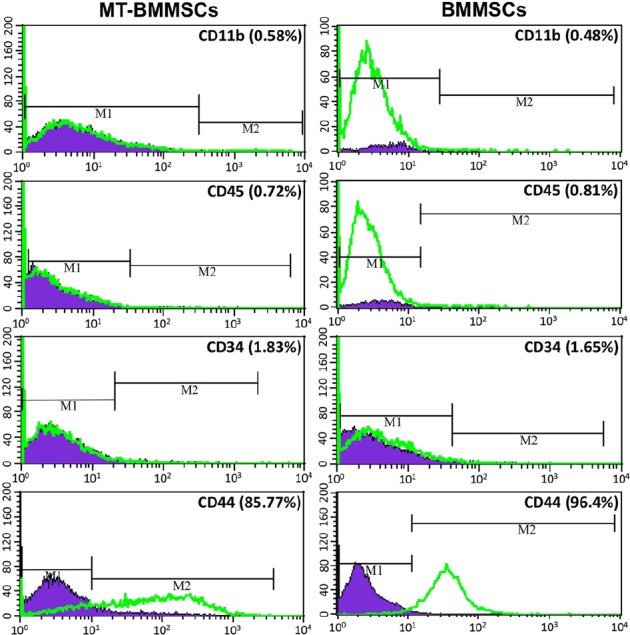
Immunophenotypic characterization of bone marrow-derived mesenchymal stem cells (BMMSCs). Flow cytometry analysis confirmed that both melatonin-pretreated BMMSCs (MT-BMMSCs) and BMMSCs expressed the low levels of CD11b, CD45 and CD34, but high level of CD44. CD44 had a fractionally higher expression in BMMSCs. M1, Isotype control; M2, CD markers expression level of BMMSCs

Concerning cell-surface antigens, flow cytometry analysis of both BMMSCs and MT-BMMSCs revealed an upward trend forCD44 expression with95.6±1.3% and 85.21±0.82%, whereas a downward for CD11b, 45, and 34 with 0.45±0.43% and 0.51±0.16%, 0.7±0.74% and 0.64±0.28%, and 1.47±0.51% and 1.71±0.17 (for all *P*≤0.05), respectively (Fig. 4).

### Carbon tetrachloride administration to induce fibrosis

**Fig. 5:**
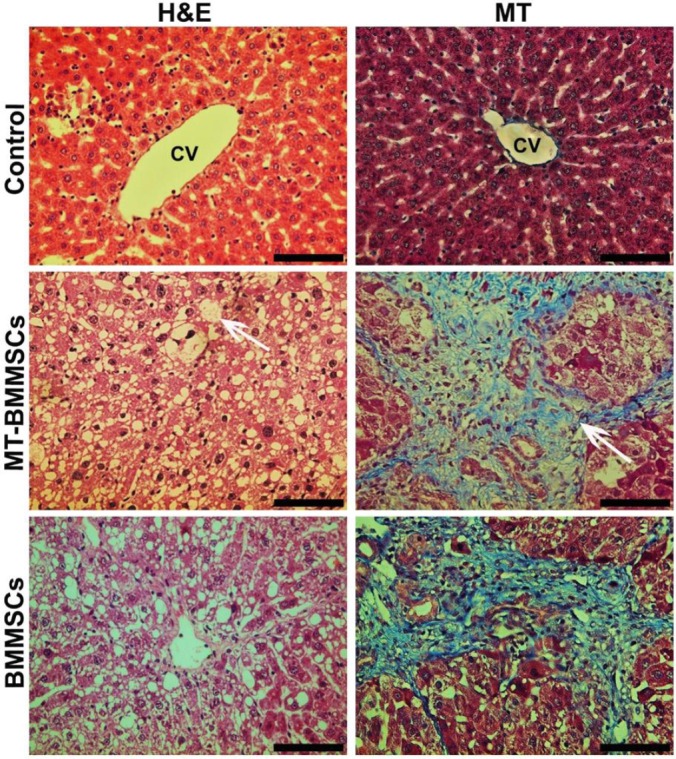
Liver sections of carbon tetrachloride-treated rats stained with hematoxylin and eosin (H&M) and Masson’s trichrome (MT). H&M staining showed tissue disruption and also high numbers of macro (arrow)- and micro-vesicular fatty change in hepatocytes of melatonin-pretreated BMMSCs (MT-BMMSCs)- and BMMSCs-infused animals. MT revealed extensive collagen deposition (arrow) in both groups. CV, central vein (Light microscope, scale bar=50 µm).

Rats subjected to CCl_4_ revealed the average weight loss of 40-60 g, as compared with normal animals. There was also a small fraction, around 5%, of mortality rates subsequent to CCl_4_ intoxication (data not shown). The evaluation of liver fibrosis was performed by two histological methods: hematoxylin and eosin and Masson’s trichrome staining. The histology of hepatic lobule and hepatocytes was normal, and slight collagen deposition was identified in the peripheral blood vessels and portal area in control group. In both MT-BMMSCs and BMMSCs infusion of CCl_4_-injected rats, the swelling of hepatocytes was observed; meanwhile, significant vacuolar degeneration was noticed in hepatic fatty tissues. Widespread necrosis and ballooning degeneration were seen in the majority of hepatocytes. Infiltration of inflammatory cells was found in the portal area and interval of fibers. Significant fibroplasia was observed in the collagen fibers located in the parenchyma of the two groups (Fig. 5).

### Homing assay

#### Fluorescent microscopic study

**Fig. 6:**
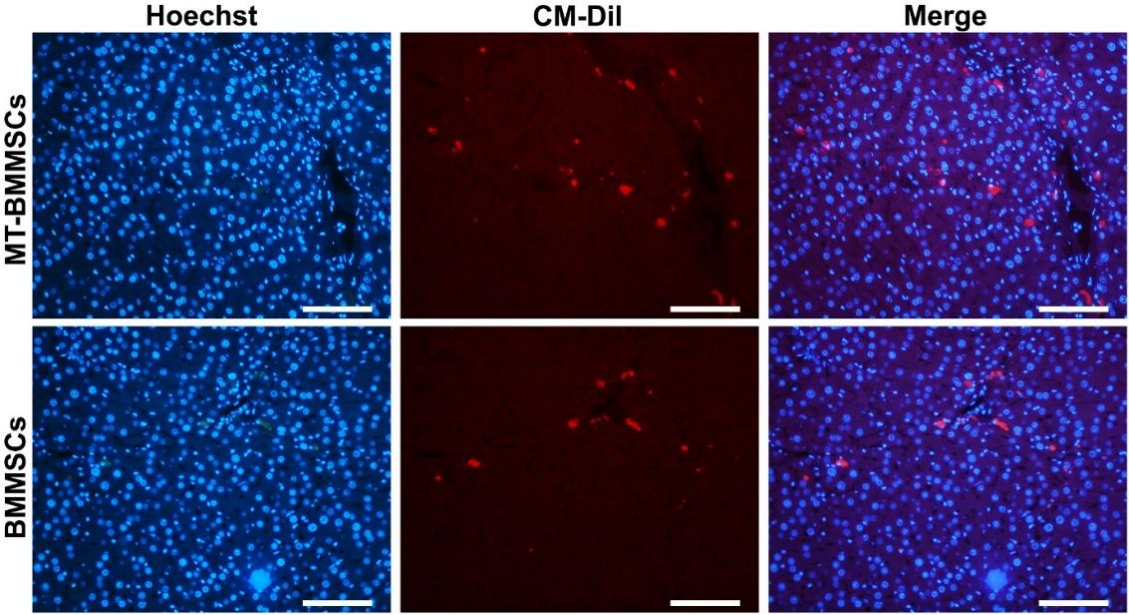
*In vivo* homing of CM-Dil-labelled bone marrow-derived mesenchymal stem cells (BMMSCs) with or without melatonin preconditioning toward tetrachloride-injured liver. Using CM-Dil (red), to track the fate of the transplanted BMMSCs, the high numbers of red stained cells were identified in melatonin-pretreated BMMSCs (MT-BMMSCs) group, as compared with not-treated BMMSCs. Nuclei were labeled with Hoechst (fluorescent microscope, scale bar=100 µm).

**Fig. 7:**
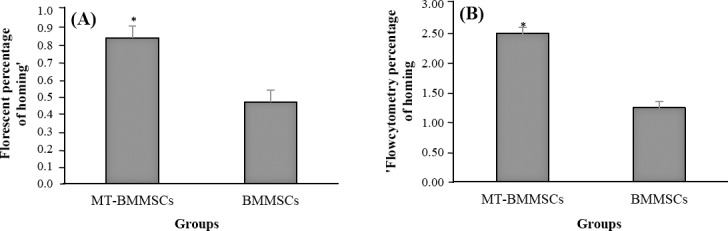
Quantifying the percentage of CM-Dil-labelled bone marrow-derived mesenchymal stem cells (BMMSCs) homing into tetrachloride-treated liver. Values are the means±SD of eight different animals. A) Florescent micrograph quantification was determined by counting the labelled-BMMSCs, which have homed to the liver by using imageQuant software. Melatonin-pretreated BMMSCs (MT-BMMSCs) showed a significant higher rate of homing (*P≤0.001); B) The total number of BMMSCs homed to the liver through the mentioned formula, based on flow cytometry data, was also significantly revealed the higher number for MT-BMMSCs (*P≤0.01).

For *in vivo* cell tracking, BMMSCs, with or without melatonin incubation, were labelled with CM-Dil, and then transplanted into CCl_4_-injured rats. Following melatonin pretreatment, labelled BMMSCs displayed much high numbers of fluorescence. On the other hand, the number of detectable CM-Dil-labelled cells in non-incubated cells was markedly smaller than incubated cells (Fig. 6). For quantifying the number of homed cells, ImageQuant software was used. The proportion for MT-BMMSCs-injected animals rose by almost 2-fold at 0.84±0.07, as compared with BMMSCs with 0.47±0.03 (*P*≤0.001) (Fig. 7A).

#### Flow cytometry

**Fig. 8:**
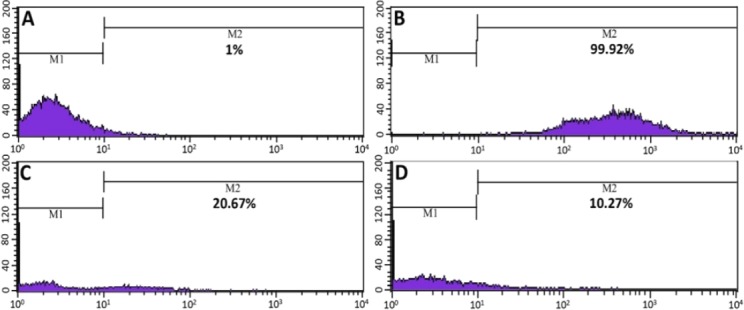
Flow cytometric assessment of bone marrow-derived mesenchymal stem cells (BMMSCs) homing. A and B showing non-injected BMMSCs: A) Unlabeled cells; B) CM-Dil-labelled cells; C) Melatonin-pretreated BMMSCs; D) Untreated cells. C and D show the percentage of red-stained CM-Dil-labelled injected BMMSCs in 1 ml homogenized liver sample. M1, Isotype control; M2, Labelled BMMSCs

The evaluation of CM-Dil-labelled BMMSCs homing was performed via flow cytometry. In comparison to not-exposed BMMSCs (Fig. 8A), BMMSCs exposed to CM-Dil showed a high percentage of labeling (Fig. 8B). Seventy two hours after transplantation of melatonin-pretreated cells, 1 ml homogenized liver sample represented 20.54±0.4% CM-Dil-labelled cells (Fig. 8C), which was approximately 2-fold as opposed to untreated cells with 10.24±0.2% (Fig. 8D). Determination of the formula number of homed cells from the total 1.5×10^6^ injected cells was carried out based on the earlier mentioned. As shown in Figure 7B, the mean of 2.5±0.2% from the whole injection was belonged to the pretreated cells. In contrast, the number for untreated cells was 1.25±0.1%. The discrepancy between the two groups was significant (*P*≤0.01).

## DISCUSSION

In the present investigation, the effect of melatonin preconditioning on homing capability of BMMSCs into CCl_4_-induced hepatic fibrosis has been surveyed. Our findings disclose the high purity of BMMSCs in multipotency and immunophenotypic features with no conspicuous change between BMMSCs and MT-BMMSCs, except for CD44 that had slightly higher expression in non-treated cells. This high purity is beneficial for using BMMSCs in therapeutic approaches[1]. Similarly, the results of the present study pointed out no remarkable difference in the extent of fibrosis as well as in liver architecture among the two groups, which may relate to the short-time period of cell exposure.

As a part of host repair and defense, homing reveals the integration and functional efficiency of exogenously injected stem cells in target organ[1], Which exerts through signals of chemokines, growth factors and adhesion molecules[16]. Chemokine stromal-derived factor-1 (SDF-1) and its receptor, G-protein-coupled C-X-C chemokine receptor type 4 (CXCR4), play a pivotal role in MSCs homing[17] so that SDF-1 has a key regulatory role in stem/progenitor cell trafficking[18]. SDF-1/CXCR4 axis expressed constitutively in liver, and the targeting MSC migration to the site of injury exert through interaction in this axis. Furthermore, in response to tissue damage (i.e. toxic agent exposure), the level of SDF-1 expression will be increased in that organ[17]. Thus, it can be assumed that CCl_4_ intoxication promotes SDF-1 expression in liver for allowing the BMMSCs homing to some extent.

Here, we verified the enhancement of homing in transplanted BMMSCs under pretreatment with melatonin. To illustrate this, for better BMMSCs homing into liver, transforming growth factor beta 1 (TGF-β1) signaling inhibition and restoration of the matrix metalloproteinase (MMP)/tissue inhibitor of MMP balance are of importance[19]. It has been claimed that both SDF-1/CXCR4 interaction and MMP-9 seem to be essential for stem cell homing potential; the latter degrades extracellular matrix components[20] and activates TGF-β[21]. Consistently, in the case of cerebral ischemia, elevation in the level of MMP-9 via glycogen synthase kinase-3β suppression caused an increase in CXCR4 expression for subsequent mediating BMMSCs migration[1]. Besides, in the liver fibrosis model, infused bone marrow-derived cells migrate to the injured liver through the expression of MMP-9[22]. On the contrary, melatonin administration in pharmacologic dosage has a negative effect on MMP-9 by its inhibition through suppressing nuclear factor-κB. Nuclear factor-κB pathway induces MMP-9 expression[23] and also inhibits TGF-β via down-regulating TGF-β/Smad signaling[24]. However, the incubation of MT-BMMSCs to the culture fibroblasts was followed by the increased expression of melatonin receptor 1-MMP-9 and decreased tissue inhibitor of MMP-2[13]. MMP-9 secretion is induced by protein kinase Cζ[25], which in turn is known to increase nuclear factor-κB activity[26] and reinforce CXCR4 expression[25]. Therefore, we assume that melatonin could have opposite functions in pharmacologic and precondition-ing dosage. It might also be an indirect relation between melatonin preconditioning and the mentioned axis, although it highly demands further studies. In addition, the receptor-mediated effects of melatonin on MSCs exert through the enhancement of survival[9] or the stimulation of engraftment[11]. For the latter function, melatonin receptor 2 is involved whereby infused cells are targeted to the injured site[11]. This is not the solely contribution of this receptor, as it also acts in cell differentiation[27]. Overall, considering this point that both melatonin receptors are expressed in BMMSCs[9], melatonin receptors/matrix enzymes interaction might be a reason for higher homing of the pretreated group, and probably in a dose-dependent manner one function of melatonin will be superior by the other.

Collectively, the data of the current study suggest that melatonin could be considered as a preconditioning agent for promoting homing potential of BMMSCs. Therefore, this strategy may represent a novel and beneficial applied approach for improving targeted stem cell therapy of liver fibrosis.
